# On human self-domestication, psychiatry, and eugenics

**DOI:** 10.1186/1747-5341-2-21

**Published:** 2007-10-05

**Authors:** Martin Brüne

**Affiliations:** 1 Department of Psychiatry, University of Bochum, LWL Hospital, Germany

## Abstract

The hypothesis that anatomically modern *homo sapiens *could have undergone changes akin to those observed in domesticated animals has been contemplated in the biological sciences for at least 150 years. The idea had already plagued philosophers such as Rousseau, who considered the civilisation of man as going against human nature, and eventually "sparked over" to the medical sciences in the late 19^th ^and early 20^th ^century. At that time, human "self-domestication" appealed to psychiatry, because it served as a causal explanation for the alleged degeneration of the "erbgut" (genetic material) of entire populations and the presumed increase of mental disorders.

Consequently, Social Darwinists emphasised preventing procreation by people of "lower genetic value" and positively selecting favourable traits in others. Both tendencies culminated in euthanasia and breeding programs ("Lebensborn") during the Nazi regime in Germany. Whether or not domestication actually plays a role in some anatomical changes since the late Pleistocene period is, from a biological standpoint, contentious, and the currently resurrected debate depends, in part, on the definitional criteria applied.

However, the example of human self-domestication may illustrate that scientific ideas, especially when dealing with human biology, are prone to misuse, particularly if "is" is confused with "ought", i.e., if moral principles are deduced from biological facts. Although such naturalistic fallacies appear to be banned, modern genetics may, at least in theory, pose similar ethical problems to medicine, including psychiatry. In times during which studies into the genetics of psychiatric disorders are scientifically more valued than studies into environmental causation of disorders (which is currently the case), the prospects of genetic therapy may be tempting to alter the human genome in patients, probably at costs that no-one can foresee.

In the case of "self-domestication", it is proposed that human characteristics resembling domesticated traits in animals should be labelled "domestication-like", or better, objectively described as genuine adaptations to sedentism.

## Introduction

The term "domestication" refers to a goal-directed process through which humans have changed physical features of plants and animals by replacing natural through artificial selection to adapt these species to specific human needs. In animals, domestication-associated changes also include behavioural characteristics, which, above all, have led to a reduction of aggression and an increase of "tameness" [1]. At least since Darwin's pioneering work on domestication [2], biologists have controversially debated whether several aspects of domestication-induced traits in animals could similarly be present in humans, and this issue has recently been reconsidered [1,3]. Even earlier, however, philosophers have been plagued with the question of man's place in nature. Jean-Jacques Rousseau (1755), for instance, had argued that "civilised" living conditions would have negative consequences, subsumed under the term "degeneration" [4]. Conversely, in the 1940s, the German philosopher Arnold Gehlen proposed a self-domestication theory of homo sapiens, according to which domestication would, on one hand, induce biological maladaptedness through abandoning natural selection, but, on the other hand, open new prospects for cultural development [5]. Similarly, recent humanism has highlighted the positive aspects of a presumed human domestication such as to prevent "brutalisation" of human societies (comment in [6]).

Whereas philosophers have extensively discussed putative effects of human self-domestication in terms of moral values, by the turn of the 20^th ^century psychiatrists became interested in the hypothesis of human self-domestication, because it seemingly provided a causal explanation for what was perceived as signs of degeneration of the human genepool ("erbgut") [7]. In 1857, the French psychiatrist Benedicte Morel sought to introduce objective measures in support of the concept of "degeneration", suggesting that subtle physical abnormalities would indicate the deterioration of mental health and also account for delinquent behaviour, because such deviations would be most prevalent in mentally ill and criminals [8]. Indeed, by the turn of the 20^th ^century, with increasing biologising of psychiatry, leading professionals were concerned about the seemingly rising number of hospitalised patients and searched for biological explanations, leaving aside social factors [9]. Hence, the hypothesis of the domestication of man was welcome, and, in light of the then prevailing cultural pessimism and upcoming eugenic idealism put forth by August Forel and Alfred Ploetz [10], readily adopted as rationalisation of a host of unresolved questions in psychiatry and related social issues. It is perhaps not exaggerated to state that this one-sided biological view of mental disorders and handicaps also contributed to what followed in Germany under the Nazi regime.

Albeit modern human biology may be largely free of moral allegations, there seems to be a need for discussing the possible impact of biological findings and hypotheses on contemporary conceptualisations of mental health and treatment options of psychiatric disorders. This premise is based on the fact that biological ideas have always been at risk of socio-political misuse, and on the concern that the advent of new genetic techniques may be tempting to "improve" human genetic material and eliminate unwanted traits, part of which could erroneously be attributed to human self-domestication.

In this article, I shall (1) deal with the biological evidence for human self-domestication and the historical development of the idea, including its entanglement with political opportunism during the Nazi epoch in Germany; (2) outline how and why the self-domestication hypothesis was adopted by leading (German) psychiatrists, and possibly contributed to positive and negative selection programs during the Third Reich in Germany; (3) finally argue that the debate between philosophy, biology, and other medical sciences including psychiatry necessitates a common language for further interdisciplinary exchange of ideas, as well as awareness of the dangers of naturalistic fallacies.

## Human self-domestication – the development of an idea

Charles Darwin was the first to systematically examine biological changes in species under artificial breeding conditions. Even though he did not refer to the question of human self-domestication in his two volumes on *Variations of Animals and Plants under Domestication *[2], Darwin proposed clear definitional criteria for the process of domestication. He emphasised (1) that the domestication of animals is more than taming, (2) that it represents a goal-oriented process for human purposes, (3) that the variability of physical and 'mental' characteristics is greater in domesticated species than in their wild ancestors, including the occurrence of dwarfism and gigantism, (4) that the behavioural plasticity and educability of domesticated species is greater, and (5) that the brain size of domesticated animals is smaller than that of their wild ancestors'.

In spite of these unequivocal definitional criteria, Darwin was remarkably vague regarding the possibility that humans could have undergone domestication. In *The Decent of Man *[11], he wrote the following (the most critical phrases are highlighted in italics by the author): "It is, nevertheless, an error to speak of man, even if we look only to the conditions to which he has been exposed, as 'far more domesticated' (Blumenbach 1865) than any other animal. ... In another and much more important respect, man differs widely from any strictly domesticated animal; for *his breeding has never long been controlled*, either by methodical or unconscious selection. No race or body of men has been so completely subjugated by other men, as that certain individuals should be preserved, and thus unconsciously selected, from somehow excelling in utility to their masters. *Nor have certain male and female individuals been intentionally picked out and matched*, except in the well known case of the Prussian grenadiers;" (p. 29) ... By contrast, in another paragraph Darwin stated: "We might, therefore, expect that civilized men, who in one sense are highly domesticated, would be more prolific than wild men. It is also probable that the *increased fertility *of civilised nations would become, as with our domestic animals, an inherited character ..." (p. 45–46). With respect to brain size Darwin argued, however, that in contrast to domesticated animals the *human brain and skull has increased *over time. Nevertheless, in the chapter on human races, Darwin reiterates that "man in many respects may be compared with those animals which have long been domesticated, ..." (p. 178); and later: "With man no such question can arise, for he cannot be said to have been domesticated at any particular period" (p. 183). And finally: "With our domestic animals a new race can readily be formed by carefully matching the varying offspring from a single pair, or even from a single individual possessing some new character; but most of our races have been formed, *not intentionally from selected pair*, *but unconsciously by the preservation of many individuals *which have varied, however slightly, in some useful or desired manner" (p. 188). In summary, although Darwin did not hold a clear position concerning the possibility that domestication could have taken place in *homo sapiens*, he pointed to the fact that no scientific proof in favour of such a hypothesis existed, particularly, due to a lack of goal-directedness or conscious selection of traits. However, he also made clear that humans might share some characteristics typical of domesticated animals such as increased fertility.

In the biological literature following Darwin, the term "domestication" became increasingly poorly defined. The criterion of intentional and goal-directed selection, which according to Darwin's definition was critical for domestication, was largely replaced, at least with respect to humans, by the equation of culture and civilisation with domestication.

An extensive evaluation of the topic was put forward by Eugen Fischer in his essay on *Die Rassenmerkmale des Menschen als Domestikationserscheinungen *("The racial characteristics of man as a result of domestication", 1914) [12]. A couple of years later, Fischer became known for his publication of *Grundriß der menschlichen Erblichkeitslehre und Rassenhygiene *("Outline of human genetics and racial hygiene"), which he edited together with Erwin Baur and Fritz Lenz in 1921 [13]; all authors later became leading authorities in Nazi eugenics and supported the legalisation of sterilisation and dismantling of welfare institutions to reinstitute the laws of natural selection [10].

In his essay on the domestication of man, Fischer suggested that domestication should be defined as a condition in which "the nutrition and reproduction has been influenced over a number of generations by humans" (author's translation). In line with these greatly relaxed definitional criteria of domestication, Fischer reasoned that humankind should be considered domesticated from the beginning of its existence. Fischer considered racial differences to be the result of domestication, because "almost all characteristics of human races could be found in domesticated animals, except for the low variability of the external ear and the lack of dappling of the skin or hair." Interestingly, Fischer regarded blond hair, blue eyes, and bright skin colour of Europeans as signs of domestication-induced partial albinism, as well as, dwarfism and gigantism in some populations, racial differences concerning the disposition for obesity, temperament, character and intelligence. Even "the permanent female breast indicates domestication much like the udder of domesticated cattle" (author's translation) [12]. However, the point that "Aryans" should be carriers of outstanding signs of domestication was apparently overlooked, a point to which I will return in the discussion. Remarkably, however, the very same attitude towards domestication and racial hygiene including support of sterilisation was also found in leading Jewish scientists such as Richard Goldschmidt, who was Professor at the Kaiser Wilhelm Institute for Biology in Berlin-Dahlem [14]. Goldschmidt argued that the abandonment of natural selection and "radical extermination of the unfit" (Goldschmidt, 1933, pp. 214; author's translation) ought to be replaced by positive and negative eugenic measures (apparently, Goldschmidt later realised that the Nazi regime held an even more radical position regarding eugenics and was expatriated by the Nazis in 1935; he was appointed Professor of Genetics and Cytology at Berkeley, CA). Even anthropologist Franz Boas, who was not a racist and strongly opposed the Nazi regime, described curly hair, variation in stature and increasing or decreasing pigmentation of the skin as signs of human domestication, but was inconclusive about how much environmental and genetic factors contributed to these variations [15]. Thus, although Fischer and colleagues may, to a certain degree, have had an opportunistic interest in mixing scientific ideas with political claims, the association of acknowledging the self-domestication hypothesis with eugenic consequences during the 1930s was not only an issue for racist scientists.

In the 1920s, another, entirely independent biological concept was adopted from embryology to explain human self-domestication. The Dutch anatomist Louis Bolk (1926) [16] postulated that adult humans would resemble juvenile apes, and that the retention of juvenile characteristics of the ancestral species into adulthood of the descendant, referred to as "foetalisation" or "neoteny", could be associated with the process of domestication. For example, the zoologist Max Hilzheimer (1926/1927) argued that "the recent European should be considered the most progressively domesticated form whereas Neanderthals were much less juvenilised" (author's translation) due to the more pronounced retention of juvenile traits in anatomically modern humans compared to Neanderthals (at that time, it was not known that Neanderthals were not ancestral to anatomically modern humans) [17]. The parallel drawn between domestication and neoteny is interesting in light of the currently resurrected debate about human self-domestication (see below).

In the 1940s Nobel laureate Konrad Lorenz' published some speculations on the relation of human psychological capacities to the process of domestication. In his article *Durch Domestikation verursachte Störungen arteigenen Verhaltens *("Domestication-induced disorders of species-typical behaviour", published in 1940) Lorenz reiterated parallels between the living conditions of civilised inhabitants of metropolitan areas with domesticated animals, which he thought indicated signs of degeneration [18]. Lorenz proposed that the intensity and frequency of instinctual patterns of behaviour were altered under these conditions, leading to a hypertrophy of some instincts due to a lowered releasing threshold and to a functional disruption of species-typical behaviours. Beside the alleged domestication-associated morphological features in human beings, such as shortening of the extremities and of the base of the skull, atony of the muscles, and obesity, which he later subsumed under the term 'Verhausschweinung' (a term hard to translate that roughly compares the physical appearance of human beings with domesticated pigs), Lorenz described a domestication-associated diminished social sensitivity and a functional disruption of love, marriage, and the "copulation drive". Apart from his appallingly coarse language, which conformed to the writing style of that time, Lorenz did not refrain from discussing racial hygienic consequences such as the "extermination of ethically inferior people." Moreover, and from our perspective today virtually ridiculous, Lorenz proposed a positive selection for *Anständigkeit *(decency) and for the physical ideal of the ancient Greek.

By contrast, in his chapter on *Psychologie und Stammesgeschichte *("psychology and epistemology", first published in 1943) [19] Lorenz took over Arnold Gehlen's idea that human beings were specialised in being non-specialised. Gehlen had acknowledged Bolk's and Hilzheimer's hypotheses as scientific proofs for his thesis of man as "Mängelwesen" ("deficient being"). Following Gehlen, Lorenz highlighted man's lack of physiological specialisation while rejecting the hypothesis of deficiency. In contrast to his earlier exclusively negative approval, Lorenz now accepted the hypothesis of domestication-associated neoteny, which accounted for the positively asserted human "Weltoffenheit" ("cosmopolitanism") and persisting explorative behaviour. This was new, since he now ascribed to neoteny a variety of human behavioural and psychological features in addition to his physical characteristics. Even in his later writings, however, Lorenz stuck to his culturally pessimistic attitude, while partially backing off from his writings during the Nazi regime.

Since the 1960s, both the foetalisation and the domestication hypotheses concerning humans have been refuted by various scientists. Starck (1962), for example, criticised that Bolk's hypothesis had been so broadly accepted simply because the many problems of explaining human evolution could be resolved with apparent ease. According to Starck, hairlessness and the reduction of pigmentation of the skin were more reliably explained by chance mutations rather than by foetalisation. Moreover, the retention of juvenile characters (i.e. neoteny) did not sufficiently explain the increased variation of traits under domestication [20]. In addition, Herre and Roehrs (1971) rejected the human self-domestication hypothesis for its lack of goal-directedness and artificial selection of traits; nor was there evidence for a "wild" ancestral human species from which a domesticated *homo sapiens *should have derived. They further argued that a reduction of instinctual patterns of behaviour in human beings could also better be explained by a more sophisticated cortical control rather than domestication [21].

As with many scientific ideas, however, the hypothesis of human self-domestication has recently been revived as a possible explanation of changes of human physical traits since the late Pleistocene. These changes include the reduction of body size and decrease in skeletal robusticity, modifications in cranial and dental features including reduction in cranial capacity, shortening of the facial region of the skull and maleruption of teeth, and reduction in sexual dimorphism. In contrast to earlier biological writings, other domestication-associated features observed in animals such as an increased variation in skin colour, increasing fat storage, earlier sexual maturation and activity, and reduction in motor activity are not discussed with respect to human self-domestication in recent accounts [1]. It is indeed plausible to assume that these changes could have taken place due to the creation of an artificially protective environment after humans adopted a more sedentary lifestyle in the Neolithic period, thereby relaxing natural selection pressures.

Similarly, the idea that foetalisation and domestication could be related, has recently been highlighted in a seminal paper comparing anatomical features and behaviour of apes and humans [3]. The authors argue that changes in social structures of early humans, compared to our closest living relatives, the chimpanzee, could have favoured the selection against aggression, and that such selection was accompanied by a reduction of sexual dimorphism in humans and the retention of juvenile characteristics in body shape and behaviour. Interestingly, a parallel development has been proposed in the bonobo, which displays more neotenic physical features and is much less aggressive compared to the common chimpanzee [3].

From a biological perspective the greatest dispute with regard to physical changes in anatomically modern humans akin to domestication pertains to a slight but measurable decline of brain volume from around 1,400 cm^3 ^to roughly 1,300 cm^3^, which could be interpreted in further support of the human self-domestication hypothesis. However, this decline in stature was accompanied by a reduction in body size such that the allometric brain-body relation remains unchanged [22]. In contrast to humans, domesticated animals show a large disproportionate decline of brain size by up to 30%, especially of the sensory perceptual centres, compared to their wild ancestral species, yet no such pronounced decline has convincingly been demonstrated in any human population.

## Impact of the human self-domestication hypothesis on psychiatry

Quantifying the actual impact of the self-domestication hypothesis on psychiatry is a difficult endeavour. As psychiatry began to one-sidedly "biologise" mental disorders, including concepts of aetiology and treatment by the turn of the 20^th ^century [9], human self-domestication was readily acknowledged as the primary cause of degeneration, which allegedly had already begun to deteriorate the genepool of populations or "races". The impact of the self-domestication hypothesis can perhaps be estimated according to the influence of prominent psychiatrists on national and international developments in psychiatry, who proclaimed the validity of the degeneration paradigm, and hence, self-domestication. In the first three decades of the 20^th ^century, German psychiatry had certainly a leading role worldwide in terms of scientific competence and recognition. For example, many psychiatrists from abroad were fellows at the newly founded German Kaiser Wilhelm Institute for Psychiatry headed by Emil Kraepelin. Kraepelin and his pupil and successor Ernst Rüdin, who first headed the Genealogical Department of the Research Institute under Kraepelin's directorship, were two prominent figures who were ardent advocates of the degeneration and domestication hypotheses, and both did not make a secret of their views that domestication was the main causal factor for the alleged increasing prevalence rates of mental disorders and deterioration of the genepool. In his article *Zur Entartungsfrage *("On the question of degeneration", 1908) Kraepelin complained about a steady increase in psychiatric disorders in civilised people, while mental disorders remained comparably rare in "primitive" races [7]. Kraepelin had visited the psychiatric hospital "Buitenzorg" in Java in 1904 and recognised that the "paralysis of the insane" and alcoholism were rare despite a high prevalence of syphilitic infections among the Java people. This, he reasoned, would indicate a greater resistance against diseases in these peoples compared to inheritable germ lesions acquired by the forces of civilisation, which ultimately would lead to an increase of degeneration insanity ("Entartungsirresein"). (Note that Kraepelin adhered to the concept of so-called "blastophtoria" (germ lesion), which reflects Lamarckian inheritance). Kraepelin now subsumed a variety of "symptoms" under the domestication paradigm, such as "weakening of viability and resistance, decreasing fertility (as opposed to Darwin's observations in domesticated animals), proletarianisation, and moral damage due to 'penning up people' (orig. German phrase "Zusammenpferchung")". The result would be "effeminacy, stunted growth, and debility" (author's translation). Kraepelin saw further threats in one-sided "breeding of psychological capacities" in civilised populations, while neglecting physical vigour and the development of the "free will", all of which for him was most evident in Jewish people. Kraepelin's language also reflected the tendency toward brutalisation and radicalisation found among many of his contemporaries. Examples are presented here in some detail (and to my knowledge for the first time translated into English) to illustrate the other side of Emil Kraepelin, who is still a highly regarded icon in psychiatry. Kraepelin was concerned, for example, that "the number of idiots, epileptics, psychopaths, criminals, prostitutes, and tramps who descend from alcoholic and syphilitic parents, and who transfer their inferiority to their offspring, is incalculable. Of course, the damage will be balanced in part by their lower viability; however, our highly developed social welfare has the sad side-effect that it operates against the natural self-cleansing of our people. We may barely hope that the degeneration-potential will be strong enough in the long term to eliminate the overflowing sources of germ lesion. ... Nevertheless, the well-known example of the Jews, with their strong disposition towards nervous and mental disorders, teaches us that their extraordinarily advanced domestication may eventually imprint clear marks on the race" (author's translation; note that Kraepelin's ideas on domestication were included in the 8^th ^edition of his "Textbook of Psychiatry", published in 1909) [23].

Kraepelin's pupil and successor as head of the Kaiser Wilhelm Institute for Psychiatric Research, Ernst Rüdin, who later was involved, together with Fischer, Baur, Lenz, and others, in the introduction of the "law of prevention of hereditary diseased offspring" ("Gesetz zur Verhinderung erbkranken Nachwuchses") greatly acknowledged Kraepelin's attitude. In a paper published in 1910 in the *Archiv für Rassen- und Gesellschaftsbiologie *(Archives of Racial and Societal Biology), which was one of the leading journals in the field of genetics and eugenics, and of which Rüdin was co-editor-in-chief, he reasoned that the medical care for the insane was a distortion of the natural laws of the survival of the fittest and that medicine would be obliged to clean the genetic pool of the *Volk *in order to prevent ongoing degeneration [24]. He deplored "alcoholics are forced to go through a cure in order to extend their lives. Self-murderers are prevented by force from killing themselves. Only the mildly psychotic or psychopathic forms may die undisturbed ... those who refrain from food intake are artificially nourished and thus brought over death, conditions of weakness, and consuming diseases ... Numerous criminals, who in former times were laid by the heels or hanged on the spot in the name of justice or by the self-help of the *Volk *(best be translated as "people", however, with a marked nationalist connotation), are now, if mentally ill, upgraded into the rank of affectionately treated sufferers, or, if criminals, simply imprisoned by virtue of law. Many so-called 'fools' formerly perished contemptibly. How many had been burned as witches? Epileptics suffocated or were forever rendered harmless if dangerous to the community." On the other hand, Rüdin feared that peoples of high value were "destroyed" as "cannon fodder in a peaceful cultural war", which led him to advocate racial hygiene in order to stop ongoing degeneration [24].

Kraepelin's and Rüdin's emphasis of degeneration and domestication as a major explanatory factor for psychiatric disorders is intimately tied to Kraepelin's nosological system, which was, to a great deal, built on the degeneration paradigm.

Although several aspects of eugenic measures – i.e. sterilisation and euthanasia, which drew heavily on the idea that the general population was threatened by increasing domestication – were shared by other leading psychiatrists, the same psychiatrists refuted Kraepelin's one-sided biological perspective on the aetiology of psychiatric disorders (e.g., [25]), and others explicitly refuted the degeneration paradigm for its scientific flaws [26]; (overview in [9]). Thus, the acknowledgement of the degeneration paradigm and its presumed underlying cause (i.e. domestication), and the support of eugenic measures were, to some extent, separate issues. Hoche, for example, together with Karl Binding, who was Professor of Law, authored one of the most appalling pamphlets entitled "Die Freigabe der Vernichtung lebensunwerten Lebens, ihr Maß und ihre Form" (best be translated as "The approval of the extermination of valueless lives, its measures and procedures") in 1922, where they outlined euthanasia of mentally handicapped individuals [27].

In any event, in light of their outstanding position and international recognition it can be argued that the impact of Kraepelin's and Rüdin's opinion can hardly be overestimated. Rüdin was highly praised as an internationally leading authority in psychiatric genetics, and invited to attend the International Genetic Congress even in summer 1939. Notably, Rüdin's work on psychiatric genetics was still cited in a textbook of medical genetics in the early 1970 (quoted from [28]).

After World War II, the self-domestication hypothesis – at least explicitly – has only patchily been mentioned in the psychiatric literature. For example, Ernst Kretschmer (1929) who had pointed to a link between domestication and breeding of intelligence in his essay on the relation of genius and psychopathology [29] abandoned these ideas in later editions [30]. More recently, Richter (1959) assumed an association between civilisation and domestication, which, among other diseases, led to an enhanced vulnerability for mental disorders [31].

Much more popular have been attempts to link psychiatric disorders with the neoteny hypothesis, however, without explicitly referring to domestication. Bemporad (1991), for instance, assumed that a lack of curiosity in "dementia praecox" (schizophrenia) might be related to a dysfunction of regulatory genes controlling neotenic traits [32].

Crow (1995) proposed a failure of neoteny corresponding to a lack of cerebral asymmetry in psychotic disorders [33]. Feierman (1994) speculated on the possible role of neoteny in the aetiology of schizophrenia, rendering "nocturnalism" a neotenic trait [34]. Likewise, Jonas and Jonas (1974) related neurotic patterns of behaviour to neoteny, assuming that the retention of archaic responses could reflect preservation of neotenic features [35]. These ideas have, however, been refuted for their vagueness and implausibility [36]. Moreover, the neoteny hypotheses were entirely disentangled from the concept of degeneration, and, beyond doubt, none of the authors has drawn the slightest moral conclusions from their scientific considerations or confused "is" with "ought".

## Discussion

The hypothesis that humans could have undergone anatomical and behavioural changes akin to domestication of animals by relaxing the forces of natural selection represents an interesting scientific hypothesis. Even though there is more evidence against than in favour of human self-domestication, at least with respect to brain size, early scientists from Darwin's times on were fascinated with questions of potential biological consequences of modern civilisation, i.e. partial abandonment of natural selection, for physical and mental well-being (here, it may be worth mentioning that the role of sexual selection has drawn much less attention with regards to effects of modern living-conditions; my personal view is that sexual selection is at least equally important as natural selection for shaping the phenotype of *homo sapiens*, and that sexual selection simply cannot be eliminated by "civilisation"). The hypothesis of human self-domestication may, however, not only illustrate how the acceptance of scientific concepts wax and wane over time, particularly if definitional criteria change, as has been the case with domestication; the main issue here is that scientific hypotheses, particularly if pertaining to human biology, are potentially at risk of misuse. The self-domestication hypothesis came up at a time when cultural pessimism and eugenic optimism prevailed even among scientists [10], and hence, was approved without further enquiry as a valid explanation for an even more scientifically vague concept, degeneration. It was probably only a short step to move on from insufficiently explored scientific ideas to moral claims, a concept shift commonly referred to as "natural fallacy", first emphasised by David Hume as early as 1739 [37]. In the case of self-domestication, the unfortunate connection between biological science and deduced moral claims was fuelled by a lack of agreement over the question how to deal with naturalistic fallacies, or unwillingness to consider the existence of a naturalistic fallacy for reasons that may have been associated with political opportunism or perhaps firm conviction of some leading scientists in the need for eugenic measures. The atrocities that followed during the Nazi regime in Germany are quite well known and summarised elsewhere (e.g., [38,39], and shall not be repeated here in detail, but several of the inconsistencies of eugenics may be worth a closer look.

Advocating negative eugenics in order to exterminate or "weed-out" domestication-induced characteristics had a long tradition in Europe and North America long before the Nazis seized power (regarding the eugenics movement in the United States of America, see for example [40,41]). On the other hand, much less is known about positive eugenics, i.e., breeding programmes to improve the genetic quality of humans, in which undoubtedly racial selection criteria were applied. By the turn of the penultimate century, Willibald Hentschel, for instance, proposed the foundation of "Mittgart" in Germany, a rural community in which 1000 women and 100 men should mate in order to renew the "Germanic race" on a polygamous basis [42,43] (a very recommendable critique can be found in Oscar Hertwig's essay *Zur Abwehr des ethischen, des sozialen, des politischen Darwinismus*, 1918) [44]. Moreover, at the beginning of the 20^th ^century, scientists even inseminated female chimpanzees with the sperm of black men in order to breed cheap working slaves. These experiments failed, however, since the inseminated chimpanzees died during their transport from Africa to Europe [45].

During the Nazi regime, human breeding programmes were put into practice under the leadership of Reichsführer-SS Heinrich Himmler. On his behalf, the "Lebensborn e. V." was founded to breed Aryans according to anthropological and biometrical criteria such as blondness, blue eyes, form of the skull, etc., but also according to "mental qualities" [46-48]. All the more grotesquely, Eugen Fischer (1914) considered blondness and blue eyes to be domestication-induced variants, and proposed these for extermination [12].

These inconsistencies notwithstanding, is there anything to learn from the example of how the hypothesis of human self-domestication was conceived in the past for current problems in relation to human biology and society? If the misuse of a controversial concept like self-domestication as scientific underpinning for eugenic measures can be, in part, attributed to a naturalistic fallacy, may this happen again with regards to modern genetics? What are the limits of gene therapy, specifically from a psychiatric point of view?

Modern evolutionary biology suggests that physical and mental characteristics of humans represent biological adaptations, albeit there may be plenty of trade-offs and design compromises. We know today that the so-called "diseases of civilization" are not the result of degeneration or domestication; rather, many disorders that supposedly fell into this category result from a mismatch between adaptation and the modern environment; in other words, culture and civilisation shape the actual phenotype. For example, obesity, some subtypes of diabetes mellitus, hypertension, and coronary heart disease probably emerge from the selection of so called "thrifty genes", which were selected because they were once helpful in the environment of evolutionary adaptedness to maintain the storage of energy during times of relative food scarcity [49-55]. Only in modern living conditions with our excessive calorie-intake have these adaptations lost their beneficial effect, at least in Western industrial societies. Thus, many modern diseases are more environmentally rather than genetically caused. Moreover, the human genome comprises much less active genes than originally assumed. Pleiotropy, a case in which a single gene influences multiple traits, and the interaction of many genes may therefore be the rule rather than the exception. Thus, it is likely that gene manipulation would have an incalculable impact on traits other than the targeted one; such problems have recently been described in animal experiments, where a gene passed into a genome of mice unexpectedly induced leukaemia [56].

In light of these considerations, can we, at all select mental traits for genetic manipulation? Negative eugenics would imply that distinct predisposing alleles ought to be eliminated from the gene pool of a population: the genes in question would perhaps be detectable in pre-implantation diagnostic screening. Which diseases should be targeted? Perhaps "Alzheimer's disease" (in this case, had the technology been available, the great philosopher Immanuel Kant would not have been born) [57]; or "depression" (we surely would have prevented the birth of Johann Wolfgang von Goethe) [58]; or last but not least "panic disorder" (in this instance, even Charles Darwin would perhaps have had fallen prey to negative eugenics) [59]. On the other hand, the potential targets of positive eugenics are equally questionable: Would, for instance, "intelligence" be a valuable trait (in most instances, the term is understood as technical intelligence)? We may speculate, however, whether we would have to deal with an increase of autistic spectrum disorders, since these disorders seem to be more prevalent in families in which the fathers and grandfathers had been technicians [60].

It is not my intention to demonise gene technology *per se*, but it should in the first place be made available to benefit those thousands of children and adults in the third world who die from malaria, tuberculosis and other infectious diseases, which further suggests that gene technology companies should promote the production of vaccines and antibiotics. Furthermore, such theoretical models of human genetic improvement disregard the impact of environmental factors in shaping the actual phenotype (note, for example, that many children found in the Nazis' breeding grounds of the "Lebensborn" after the end of World War II were developmentally retarded, probably due to deficient attachment in early childhood) [46].

## Conclusion

Man, like all other living beings, is characterised by specific and unique evolved design compromises, such that most physical and psychological features have costs and benefits [61]. Processes akin to the domestication of animals may, to some degree, have been involved in shaping human nature, but this assumption depends, above all, on the definitional criteria of the term "domestication". We ought to accept our biological heritage and not be enticed into mistaking biological and technological feasibility for scientific progress. Except for the fact that a manipulation of the human genome would "benefit" only a very small minority of the world population, it is in my opinion more advisable to protect and to recreate environments worth living in, ones which take into account our biological predispositions, a claim made very early by Alfred Russel Wallace, who was a grim opponent of eugenics [62]. Evolution does not know a higher, or a better, not even in terms of humankind.

In this article, the process of domestication has served as an example of how science can be intertwined with philosophical or socio-political issues, known as naturalistic fallacy. In light of the negative connotation of the term "domestication", "domestication-like" might be a better term for human characteristics that resemble domestication-induced traits in animals. Alternatively, the term might be replaced by the neutral expression of "adaptive changes to sedentism". This may, at first sight, be only a matter of wording and definition, but scientists should be sensitive that scientific concepts can be associated with values. Similarly, philosophers should not seek for an "is" to support their claims for "ought", a problem that may be referred to as "reversed naturalistic fallacy". Beyond all discussion of the validity of the self-domestication hypothesis, the example may support the necessity for an interdisciplinary exchange of ideas between the life-sciences and philosophy to facilitate an open dialogue.

## Competing interests

The author(s) declare that they have no competing interests.
